# Influence of Seaweeds on the Quality of Pasta as a Plant-Based Innovative Food

**DOI:** 10.3390/foods11162525

**Published:** 2022-08-21

**Authors:** Andrea Ainsa, Adrián Honrado, Pedro Marquina, José A. Beltrán, Juan Calanche

**Affiliations:** Instituto Agroalimentario de Aragón-IA2-(Universidad de Zaragoza-CITA), Miguel Servet. 177, 50013 Zaragoza, Spain

**Keywords:** pasta, seaweed, quality, technological properties

## Abstract

This study evaluated the effect of the incorporation of seaweed on the physicochemical and technological quality of pasta. For this purpose, enriched wheat pastas from different seaweeds (sea lettuce—*Ulva lactuca*, nori—*Porphyra tenera*, and wakame—*Undaria pinnatifida*) were made and compared with durum wheat pasta as a control treatment. Firstly, optimal cooking times were established by visual and instrumental methods. Then, the technological properties of weight gain (WG), swelling index (SI), cooking losses (CL), and moisture (H%) were determined. Protein and fiber analyses, texture profile analysis (TPA), and color measurements were also performed to evaluate the physicochemical properties. Overall, enriched pasta with seaweed revealed slightly shorter optimal cooking times than control pasta. Texture properties were also modified, with a lower value of hardness, and higher values of adhesiveness and resilience. However, due to the low percentages of seaweed (3%), noticeable effects were not appreciated. Moreover, color variations of enriched pasta were relevant due to the difference among seaweeds. Nonetheless, these additions increased the protein content and soluble fiber in these foods. In conclusion, pasta enriched with marine ingredients improved this nutritional profile, and the changes in technological properties did not have a major impact on the product quality.

## 1. Introduction

New foods based on plant-based studies are becoming more common every day. Lifestyle changes, e.g., diet, can be important and powerful tools in treating chronic illness or simply maintaining good health. Nowadays, plant-based diets have become mainstream among people partly because the advantages have been well researched and healthcare practitioners recommend this way of eating as many have seen satisfactory results from their patients. Moreover, it can be advantageous for physicians to recommend and support plant-based eating to achieve optimal health outcomes and possibly minimize the need for procedures, medications, and other treatments [1].

In this sense, the term Novel Food can refer to a newly developed, innovative food produced using new technologies and production processes, as well as food which is or has been traditionally eaten outside the EU. Novel Food is defined according to European legislation as “food that had not been consumed to a significant degree by humans in the EU before the first Regulation on novel food in 1997”. Novel Food in the European Union must be safe for consumers, properly labeled and, if this novel food is intended to replace other food, it should still be nutritionally advantageous for the consumer [2].

Seaweeds represent an important group of these Novel Foods that show many advantages for health, which contribute to increasing their use as food. Algae are known as “aquatic plants” since they have a very similar appearance, as they are also autotrophic microorganisms with the ability to carry out photosynthesis, but with the difference that they present a simpler structure with little or no cell differentiation and with complex tissues, which are called talophytes [3]. Seaweeds stand out for their great nutritional value, being rich in protein, fiber, polyunsaturated fatty acids, vitamins, and minerals while having a low energy value. Those of the greatest interest to the functional food industry are bioactive compounds, such as polysaccharides and proteins, polyphenols, pigments such as carotenoids, and the ω-3 fatty acids [4]. It is a fact that one of the main advantages of algae is its lipid fraction rich in ω-3 polyunsaturated fatty acids of high molecular weight, especially EPA (eicosapentaenoic acid) and DHA (docosahexaenoic acid), showing an ω-6/ω-3 ratio that is ideal for reducing this ratio in foods where it can be incorporated as a functional ingredient [5]. Furthermore, seaweeds are a rich source of micronutrient compounds, such as vitamins (e.g., vitamin A, B1, B2, B3, B6, B12, C, D, E, pantothenic acid and folic acid, sterols) and minerals (e.g., calcium, magnesium, potassium, iodine, sodium, phosphorus, nickel, chromium, selenium, iron, zinc, manganese, copper, lead, cadmium, mercury, and arsenic). Moreover, seaweeds are known to be one of the best natural sources of iodine [6].

As can be seen, marine products represent a great source of compounds perfect to be used as functional ingredients [7]. Therefore, the combination of this group of foods of great nutritional value with pasta, a food product of great acceptability with a high energy value, makes them perfectly compatible to result in food enriched with beneficial properties to health. Seaweeds have been used in many products such as meat, fish, bakery products, dairy products, vegetables, fruits, and pasta [6]. Depending on their protein composition and functional characteristics, algae offer the possibility to be incorporated into food, especially meat products and others based on cereals, to allow maintaining or improving their sensory and nutritional quality by incorporating pigments, functional proteins, and polyunsaturated fatty acids, mainly [3]. Considering the wealth of information available about the functional properties of seaweed and seaweed extracts, it will be interesting to review how effective these compounds have been when they are incorporated into several food products [6]. 

Due to the abovementioned, this work focused on the use of macroalgae, which in turn are divided into *Chlorophyceae*, green algae containing chlorophyll a and b, *Phaeophyceae*, brown algae that contains pigments such as chlorophyll a and c, carotenoids and xanthophyll, typical of marine ecosystems, and *Rhodophyceae*, red algae that contains red pigments such as phycoerythrin [8]. One of the most consumed green algae is Sea lettuce (*Ulva lactuca*), which was selected for this research, as well as brown algae, a group that includes the majority of edible algae, wakame (*Undaria pinnatifida*) and the popular and widespread red algae known as nori (*Porphyra tenera*) [9].

On the other hand, the quality of pasta is determined to a large extent by its technological properties, such as weight gain and hydration and cooking losses, texture, color, and sensory properties [10]. In this sense, the optimal cooking time is one of the most important parameters to be considered by consumers. This could be defined as the time required to obtain a pasta “al dente” [11]. This is the point of the highest quality in cooked pasta, since it leaves a firm and resistant texture, without stickiness on the surface and with few cooking losses. In addition, processing parameters, the chosen raw material, with its size characteristics of the particles (granularity), the quantity and quality of its proteins, mainly due to the gluten, and the technological properties of starch constitute the conditions to be taken into account to guarantee quality “al dente” pasta [12]. Besides, the color of the pasta is another key sensory attribute to consider, being positively valued as an attractive yellow caused by the presence of carotenoids present in wheat [13].

The incorporation of algae in pasta to improve its nutritional and healthy properties implies that the technological properties of this food will be affected. For this reason, it is extremely important to study the modifications that it entails, with the purpose of the final quality of this new functional food being suitable for consumers. This study aimed to evaluate the effect of the physicochemical and technological properties in enriched pasta with seaweeds in comparison with traditional durum pasta.

## 2. Materials and Methods

### 2.1. Materials

To make all developed pasta, semolina of durum wheat (*Triticum durum*) with a quality certificate (Innova Obrador S.L., Zaragoza, Spain) was used. Regarding the seaweeds used (*Ulva lactuca*, *Porphyra tenera*, and *Undaria pinnatifica*), all of them were organically produced and dehydrated (PORTO-MUIÑOS S.L., La Coruña, Spain). The shiitake mushroom extract (*Lentilula edoles*) was supplied by the company Coala S.L. (Alfaro, Spain).

### 2.2. Pasta-Making

Three types of pasta were manufactured and compared with a “control” composed of wheat semolina and water. All kinds of pasta were made with durum wheat semolina, 3% of seaweed, and 0.1% of shiitake as a flavoring, with enough water required for their preparation taking into account the proportion of solid and liquid ingredients shown in other studies [14,15]. Pastas were made in an extruder (Bottene, Mod. Lillodue 14057CE, Marano Vicentino, Italy) in macaroni format, which was selected because it is one of the most consumed in terms of short pasta and there is no information in the literature that reflects the quality of this format with the incorporation of seaweeds as a new ingredient. To prepare pasta, firstly, the dry ingredients were mixed, and then water was added slowly. Secondly, the extrusion of pasta took place. The final product was packaged and stored frozen (−20 °C). 

### 2.3. Protein and Fiber Determination

Protein content was determined by the Kjeldahl method [16]. Pasta (1 g) was digested in a 98% sulfuric acid solution (12 mL) in the presence of catalysts and heat (1 h at 420 °C) in a digester (VELP Scientifica, DK 6 Heating Digester Kjeldahl, Usmate, Italy). The samples were allowed to cool until reaching 50 °C. The digested mixture was neutralized with NaOH solution (30%) and distilled over a known volume (30 mL) of 3% boric acid using a distillation unit (VELP Scientifica, UDK 129, Usmate, Italy). Finally, the distillate was titrated in an automatic titrator (SI Analytics, Titroline 5000, Mainz, Germany) with HCl to determine the nitrogen content in the sample. To calculate the percentage of protein, the conversion factor used was 5.5, according to the recommendations for this type of food [17]. Regarding fiber analysis (Dietetic Total Fiber and Soluble Fiber), an enzymatic method was carried out [18]. All samples were milled, and the fat extraction was carried out with petroleum ether. The sample (1 g) was weighed and 25 mL of sodium phosphate buffer, pH 6.0, was added. After adjusting the pH according to steps found in the literature [18], the samples were filtered and washed with distilled water. The filtrate was the soluble fiber, which was filtered after adding 400 mL of ethanol. Then, it was dried at 105 °C and incinerated at 550 °C for 5 h. Blank was obtained with the same procedure, but without a sample. To calculate the percentage of soluble fiber, the following expression was used:

(1) $$\%\;soluble\;dietary\;fiber=\frac{D-I-B}{W}\cdot 100$$

where *D*: weight after drying; *I*: weight after incineration; *B*: weight blank; *W*: sample weight.

### 2.4. Optimal Cooking Times

This determination was carried out in two ways, applying a visual method and an instrumental method using a texturometer (ANAME Instrumentation Scientific, mod. TA-XT2i, Pamplona, Spain) with a flat Warner–Bratzler probe. This test consisted of measuring the hardness of the cooked pasta. The measurements were performed from 30 to 180 s and 7 times for each cooking time. The conditions used in our previous studies were repeated [15].

According to the AACC 66-50.01 method or a visual method [19], a certain number of samples (≈3–4) were extracted every 30 s during cooking. They were compacted using two transparent methacrylate plates. The time was estimated visually when the white core of the pasta disappeared.

### 2.5. Texture Profile Analysis -TPA-

A texture profile analysis (TPA) of pasta allowed the determination of different texture properties: hardness, adhesiveness, springiness, gumminess, chewiness, resilience, and fracturability. For this purpose, a texturometer (ANAME Scientific Instrumentation, mod. TA-XT2i, Pamplona, Spain) with a cylindrical, flat aluminium probe was used to perform two compression cycles with a decompression time of 20 s. The following conditions were established: test speed: 2 mm/s; sample deformation: 75%; force threshold: 10 g.

### 2.6. Pasta Color

Color measurements were determined in the optimal cooking time of each pasta. For this purpose, two samples were arranged together and flattened between two methacrylate plates to be representative and homogeneous. Each sample was repeated 3 times using a colorimeter (Minolta, CM-2002, Osaka, Japan). The following color parameters were recorded: L* (brightness), a* (redness), and b* (yellowness). The total color difference (∆E), from a commercial durum wheat pasta as a reference, was calculated using the following formula:

(2) $$\Delta E=\sqrt{{\left(\Delta L^{*}\right)}^{2}+{\left(\Delta a^{*}\right)}^{2}+{\left(\Delta b^{*}\right)}^{2}}$$

where: ΔL *=* L* Seaweed pasta − L* Standard pasta; Δa = a* Seaweed pasta − a* Standard pasta; Δb *=* b* Seaweed pasta − b* Standard pasta.

### 2.7. Technological Properties

#### 2.7.1. Weight Gain and Swelling Index

These properties were determined by the procedure described in Cleary and Brennan, 2006 [19]. Pasta (3 g) was cooked in 180 mL of water during the optimal cooking times. Then, it was cooled in 100 mL of cold water and dried with absorbent paper. Finally, it was weighed. The weight gain was expressed as a percentage of the total weight, according to the following formula:

(3) $$Weight\;gain=\frac{Cooked\;pasta\;weight-Raw\;pasta\;weight}{Raw\;pasta\;weight}\cdot 100$$

To determine the swelling index, pasta was placed in an oven at 105 °C until a constant weight was reached after 24 h. Then, the samples were tempered in a desiccator and weighed. The swelling index was obtained with the following expression:

(4) $$Swelling\;index=\frac{Cooked\;pasta\;weight\;\left(g\right)}{Dried\;pasta\;weight\;\left(g\right)}$$

#### 2.7.2. Cooking Losses

For the determination of cooking losses, 3 g of each pasta was cooked in 180 mL of water during the previously established cooking times [20]. The cooking water was collected in crucibles which were placed in an oven at 105 °C until evaporation was achieved. The dry residue was weighed and calculated as a percentage of the total weight of the pasta before cooking.

#### 2.7.3. Moisture

Moisture was determined by a gravimetric method. To do this, pasta was weighed and heated at 105 °C to reach a constant weight (24 h). Then, pasta samples were kept in a desiccator for 1 h at room temperature. Finally, they were weighed again. The equation to determine the moisture content is:

(5) $$Moisture\;\left(\%\right)=\frac{Raw\;pasta\;weight-Dried\;pasta\;weight}{Raw\;pasta\;weight}\cdot 100$$

### 2.8. Statistical Analysis

Statistical analyses of all results obtained were performed with the Microsoft Excel XLSTAT statistical software (Version 2016, Addinsoft^©^, Paris, France). First, univariate data analyses were performed to check their normality and locate those data that were atypical. In some cases, bivariate analyses were carried out, determining coefficients of Pearson’s correlation. In the general data obtained, an analysis of variance (ANOVA) was carried out, as well as a multiple comparison test (Fisher LSD) with a confidence level of 95%. In addition, all data obtained, with exception of color measurements, were included in the overall analysis using a Principal Component Analysis (PCA) as an exploratory study that establishes, through a confidence test (Bootstrap graphics), the relationships among different parameters assessed in the research.

## 3. Results and Discussion

### 3.1. Protein and Fiber Content

**Figure 1:**
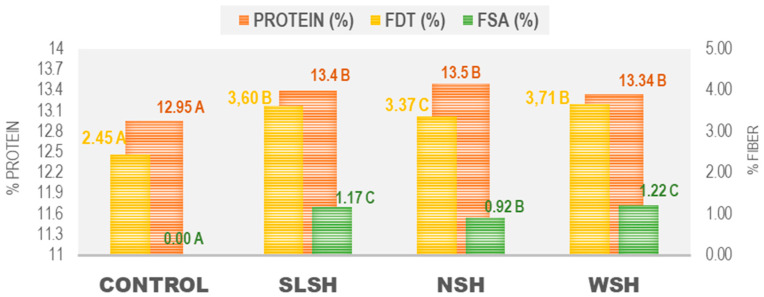
Protein content and fiber (%) in the different types of pasta. FDT: Total dietary fiber; FSA: Soluble fiber; SLSH: Sea lettuce shiitake; NSH: Nori shiitake; WSH Wakame shiitake. Different letters indicate significant differences (*p* < 0.05) among all the pastas.

According to the Spanish regulation [21], the minimum content of protein referred to as a dry substance in fresh pasta is 9.5%. As shown in Figure 1, the four pastas met this requirement, exceeding those values. The control pasta was the one with the lowest protein content, presenting significant differences (*p* < 0.05) with the pasta enriched with algae.

The kinds of developed pasta were compared to each other, and significant differences among seaweed used were not detected. Lower protein content was established in durum pasta (*p* < 0.05). The amounts of protein found were consistent with data reported in the literature, where Nori seaweed had a protein content of 29 g/100 g, while wakame and sea lettuce had 17 g/100 g [22]. In terms of total dietary fiber, a significant difference (*p* < 0.05) was only found for durum pasta (<3%), whereas for the soluble fiber content, the highest content (*p <* 0.05) was established for sea lettuce and wakame. For its part, nori showed the lowest value, and as expected the durum paste did not provide any results for this nutrient. Our results about fiber composition are in agreement with Gupta and Abu-Ghannam (2011), who claimed that the high content of fibers and mineral elements in seaweeds advocates their use as a means to improve the fiber content and reduce the salt content of many food products [23].

### 3.2. Optimal Cooking Times

**Figure 2:**
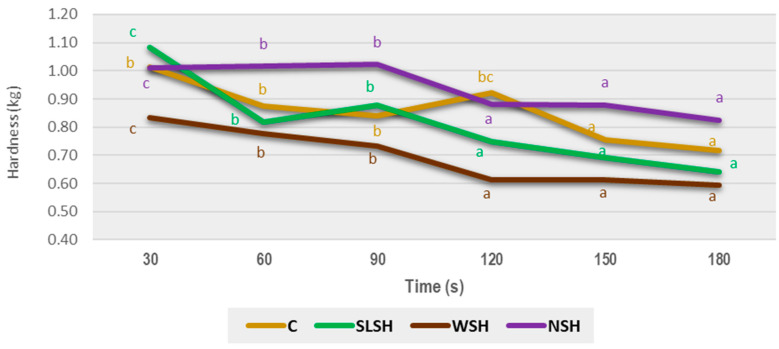
Optimal cooking time according to an instrumental method for the type of pasta assessed. C: control; SLSH: sea lettuce shiitake pasta; NSH: nori shiitake pasta; WSH wakame shiitake pasta. Different letters indicate significant differences (*p* < 0.05) among the cooking times for each kind of pasta.

The different cooking times against the hardness (kg) determined by the instrumental method are shown in Figure 2. As can be seen, the hardness decreased as the cooking time increased until it reached a maximum point from which a sharp decrease could be seen. Therefore, this inflexion point represented the optimal cooking time for pasta [24].

In all cases, the optimal cooking time was 90 s, except for the control pasta whose time was 120 s. The addition of the algae could have weakened the structure of pasta because their proteins are not able to develop a gluten network, and thus facilitate water diffusion into the gluten–protein network, decreasing the hardness of pasta [25]. This fact would imply a shorter cooking time, as observed in this research. When marine food is added to pasta, the starch content decreases and for this reason, the water required declines. The replacement of semolina indicates a decrease in the glutenin substance as has occurred in other studies [26,27]. The results obtained by the instrumental method were compared with those estimated by the visual method. These data are shown in Table 1. The values obtained by the visual method were well correlated with instrumental data (r^2^ > 0.99) in most cases, except for pasta with nori added, due to its black color, which made it difficult to appreciate the disappearance of the white nucleus. Therefore, the cooking time determined by the instrumental method was chosen because of its greater objectivity.

### 3.3. Texture Profile Analyses (TPA)

The results of the texture profile analysis of the pasta studied are set out in Table 2. It should be noted that the values were high compared with previous studies [24], so it is conceivable that, being a macaroni format, the thickness and the double layer that the cylinder forms when compressed influenced the parameters evaluated. For all pasta studied, the hardness, gumminess, chewiness and fracturability demonstrated similar behaviors, where the control always showed the highest values and the nori pasta (NSH) the lowest. Other studies pointed out that ingredients incorporated in enriched pasta could promote a weakening in the dough structure of pasta, supporting this behavior [24,28]. Nevertheless, the enrichment with algae was supposed to cause a significant increase (*p* < 0.05) in parameters such as adhesiveness, resilience, and fracturability due to the higher protein content, as has been shown in other research [29]. NSH had the highest adhesiveness in the study, being significant (*p* < 0.05) for pasta with green algae (sea lettuce and wakame). Moreover, pasta enriched with wakame (WSH) showed a similar value to control pasta. 

According to literature, the composition of algae rich in proteins produced a change in the structure of the dough of pasta, causing a weakening in this enriched pasta [28,30]. Concerning springiness, the enrichment with these marine ingredients resulted in a loss of elasticity compared with the control. These effects could be produced due to the weakening of the structure with the substitution of wheat for seaweeds [31]. Finally, the resilience was significantly (*p* < 0.05) higher in pasta with sea lettuce (SLSH) compared with the other pasta, among which significant differences were not detected. 

### 3.4. Pasta Color

**Figure 3:**
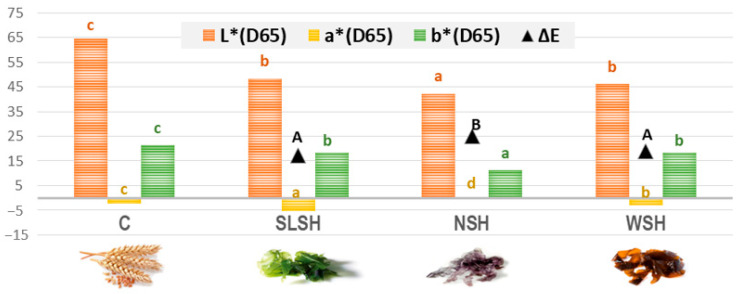
Color parameters in different types of pasta. C: control; SLSH: sea lettuce shiitake pasta; NSH: nori shiitake pasta; WSH wakame shiitake pasta. Different letters indicate significant differences (*p* < 0.05) among all the pastas for each parameter.

The parameters for the color of the different formulations of cooked pasta are represented in Figure 3. As can be seen, there were significant differences in the luminosity (L*) of the control pasta compared with the others. This was due to the common pasta (C) being yellow, the pasta enriched with sea lettuce and wakame (SLSH and WSH) being green, and the pasta with nori (NSH) being black. Therefore, the luminosity was significantly higher (*p* < 0.05) for the control and significantly lower (*p* < 0.05) for NSH.

Regarding the value of the red index (a*), the sea lettuce formulation (SLSH) presented the highest negative value, due to its green tones, followed by the wakame pasta. The formulation with nori presented the highest values due to its black color. Finally, regarding the yellow index (b*), the highest value corresponded to the control pasta, as expected, presenting significant differences (*p* < 0.001). The yellow values had the same behaviour as the luminosity.

The mean values of the color difference (∆E) show the variations of the enriched pasta compared with the control. The formulation with nori was the one that presented the greatest color change, due to the low luminosity, the decrease in the yellow index, and the increase in the red index.

Although the typical yellow pasta color, which was provided by wheat carotenoids [13], is the most positively valued, nowadays consumers are used to seeing new colors in commercial pasta. Today, pasta has a wide variety of colors due to the use of vegetable additives, which affect its typical color [10]. For this reason, although the color is modified by the algae, it should not be considered a negative attribute because this color attracts the consumer and the addition of seaweeds as a natural ingredient in pasta made it look more attractive and innovative [32].

### 3.5. Technological Properties

The data obtained from the technological properties analyzed can be seen in Table 3. The weight gain showed significant differences (*p* < 0.05) among the samples despite the low percentage of enrichment used. Likewise, the lowest value could be seen for NSH. By incorporating external ingredients into pasta, the protein matrix was modified by the new components. This largely depends on the type and quantity of proteins of the new ingredient incorporated, since the interactions with the starch will be different [33]. Owing to the algae having proteins that compete with starch for water, leaving it ungelatinized, the weight gain ratio could decrease [34].

Regarding swelling index, pasta with nori (NSH) was the only one that presented significant differences (*p* < 0.05) compared with the control. In previous studies incorporating algae into pasta, the swelling index increased with rising levels of algae in the product [35]. Differences in the water-holding capacity of seaweeds are due to their polysaccharide composition of dietary fiber fractions [36]. Brown algae (wakame) comprised higher values of fiber and, therefore, caused greater a swelling index compared with red algae (nori) [37,38]. In this study, the enrichment of algae was 3% and, therefore, the significant difference in pasta with nori was probably due to its lower fiber content and, thus, less water retention.

Cooking losses occur due to excessive swelling of the starch, which can be avoided thanks to an optimal protein structure that resists it, thus supporting the firmness of the cooked pasta [39]. Although the addition of new ingredients can weaken the protein network [34], it can also cause an interaction between the starch granules and the protein matrix, strengthening its structure, and thus, reducing cooking losses [40]. This effect could be seen in the results of this study, with pasta enriched with algae presenting lower values (*p* < 0.05). Among the enriched pasta, pasta with wakame had the lowest value due to its higher fiber content and higher water retention [37]. Despite this difference, it is necessary to highlight that minimal cooking losses were achieved, without ever reaching 3% in any of the cases, since the quality of pasta was not considered unacceptable until losses greater than 8% [34]. 

In general, moisture presented the same behavior as cooking losses. All pastas studied were around the maximum moisture limit (30%) for fresh pasta [21], but pasta enriched with sea lettuce (32%) slightly exceeded this limit. This fact could be a rehydration process due to the highly hygroscopic nature of this seaweed which is related to its soluble fiber content.

### 3.6. Global Approach of Enriched Pasta with Seaweeds Quality

**Figure 4:**
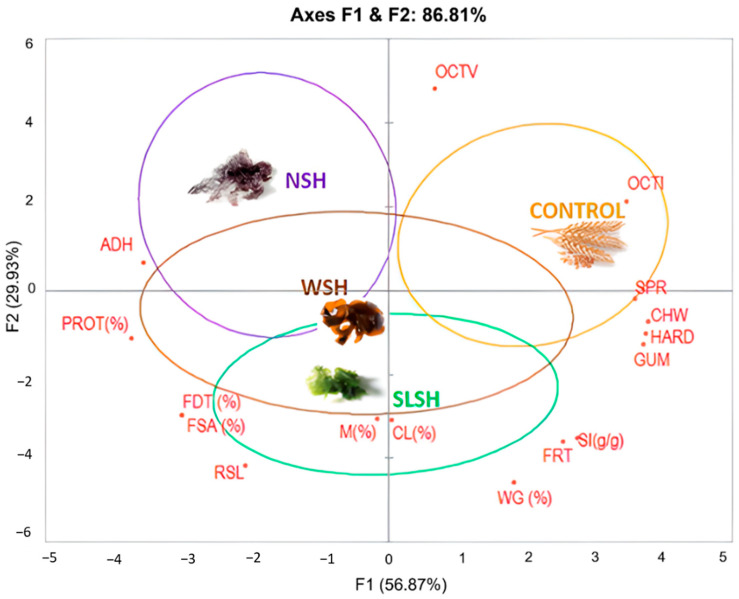
Principal Component Analysis, PCA, from TPA and technological property results in all pasta studied. SLSH: sea lettuce shiitake pasta, NSH: nori shiitake pasta, WSH wakame shiitake pasta, HAR: Hardness, SPR: Springiness, GUM: Gumminess, FRT: Fracturability, CHW: Chewiness, RSL: Resilience, ADH: Adhesiveness, M: Moisture, CL: Cooking Losses, OCTV: Visual Optimal Cooking Time, OCTI: Instrumental Optimal Cooking Time, SI: Swelling index, PROT: Protein, FDT: Total dietary fiber, FSA: Soluble fiber.

The comparative study demonstrated high correlation indices (r^2^ > 0.988) which were statistically significant (*p* < 0.05) for pasta composition, TPA, and technological properties results (Table 4). Due to the abovementioned with the purpose to achieve a better overall understanding of the study, a Principal Component Analysis was carried out (Figure 4).

The biplot obtained showed 86.81% of the total variation of the study, providing reliability and robustness to the findings of this research. The variation in the first axe (F1: 57%) was explained by the most texture parameters (hardness, chewiness, gumminess, and springiness) in contrast with pasta composition values (protein and fiber), which were in turn well associated with two texture properties (adhesiveness and resilience). In the second axe (F2 30%) the discrimination was produced due to technological parameters (CL, WG, SI, and M) located on the right side, at the bottom of the graphic in opposition to optimal cooking times (PCTI and OCTV).

The durum pasta (control) was located on the right side of the plot near the main group of texture attributes together with OCTI, as could be expected. Pastas with wakame (WSH) and sea lettuce (SLSH) were placed close to each other in the middle of the graphic. A confidence test that represents the ellipses surrounding each kind of algae (bootstrap graphics) demonstrated that both (WSH and SLSH) were similar between them, sharing characteristics, mainly texture parameters, with control pasta. However, the pasta made with green algae (SLSH) demonstrated a clear relationship with moisture (%), cooking losses (%), dietary total fiber, soluble fiber, and resilience as a texture parameter. Resilience is defined as the ability of a material to absorb energy when deformed elastically and return to its original shape upon release of load [41]. Our results are in agreement with research developed by other authors [28] who reported that the addition of seaweed powder increased the crude fiber contents of raw fresh pasta (noodles). The cooking yields of seaweed-containing noodles were also found to be higher, a fact which was attributed to the water absorption capacity of fibers and polysaccharides in the seaweed. Higher water absorption by the seaweed led to softer and spongier textural intensities in the noodles. 

Even though WSH was associated with SLSH, both had values of protein around 13% and showed higher values of adhesiveness. In the biplot, NSH turned out to be the most different compared with control and it was related to higher optimal cooking time estimated by visual assessment. According to Jyotsna et al. (2004), for good-quality pasta, the thin film of the protein network, which has to be formed by enveloping entire gelatinized starch granules, is crucial in determining the cooking quality of pasta products [42]. If this mechanism does not occur correctly, the rheological properties of the pasta could be modified, giving rise to some defects such as stickiness or gumminess.

Up to a point, the abovementioned results showed the effect of algae addition in the pasta-making process and after when it is cooking. On balance, the incorporation of seaweed extracts modified the technological properties and quality parameters of pasta. However, this alternative, a plant-based innovative food enriched with algae, represents a good strategy that will also make it popular amongst the non-seaweed-eating population.

Finally, since this work did not consider the limitations associated with any sensory characteristics (appearance, taste, texture, etc.) that algae could impart to the products in which it is used, they must be developed in future research. 

## 4. Conclusions

The addition of seaweed to pasta, in some cases, modified its physicochemical and technological properties. The incorporation of these marine ingredients significantly increased its protein content. The optimal cooking times of enriched pasta were slightly lower than control pasta. The addition of seaweed modified its texture profile, decreasing the hardness, gumminess, chewiness, fracturability, and springiness compared with the control. On the contrary, adhesiveness increased with algae enrichment. The enriched pasta group was very different from the control concerning color, with a lower lightness. Pasta with sea lettuce and wakame was green while pasta with nori was black. For this reason, the highest color variation was in pasta with nori (NSH). Finally, technological quality parameters of pasta, in general, remained similar to control pasta due to the low percentage of seaweed incorporated (3%).

## Figures and Tables

**Table 1:** Optimal cooking time for each pasta in both types of methods.

Treatment	Instrumental Method (s)	Visual Method (s)
**Control**	120	120
**Slsh**	90	90
**Nsh**	90	120
**Wsh**	90	90

SLSH: sea lettuce shiitake pasta; NSH: nori shiitake pasta; WSH: wakame shiitake pasta.

**Table 2:** Texture profile analysis at the optimal cooking time for each kind of pasta assayed.

Treatment	Hardness	Adhesiveness	Springness	Gumminess	Chewiness	Resilence	Fracturability
**Control**	14904.54 ^c^	−251.75 ^a^	0.83 ^b^	10490.02 ^c^	8660.09 ^c^	0.31 ^a^	1857.34 ^c^
**Slsh**	11953.83 ^b^	−183.86 ^b^	0.77 ^a^	8965.13 ^b^	6767.37 ^b^	0.40 ^b^	1910.54 ^b^
**Nsh**	8492.11 ^a^	−131.30 ^c^	0.71 ^a^	6610.68 ^a^	4630.46 ^a^	0.35 ^a^	993.95 ^a^
**Wsh**	11821.46 ^b^	−213.83 ^a^	0.72 ^a^	8391.13 ^b^	6057.05 ^b^	0.35 ^a^	1907.99 ^b^

Different letters indicate significant differences (*p* < 0.05) among all the pastas for each technological property.

**Table 3:** Values of technological properties for each pasta analyzed.

Treatment	Weight Gain (%)	Swelling Index (g/g)	Cooking Loses (%)	Moisture (%)
**Control**	48.76 ^a^	2.30 ^b^	0.85 ^b^	30.23 ^b^
**Slsh**	51.53 ^a^	2.32 ^b^	2.21 ^c^	32.61 ^c^
**Nsh**	43.88 ^b^	1.82 ^a^	0.80 ^b^	30.35 ^b^
**Wsh**	49.70 ^a^	2.26 ^b^	0.12 ^a^	29.12 ^a^

SLSH: sea lettuce shiitake pasta; NSH: nori shiitake pasta; WSH wakame shiitake pasta. Different letters indicate significant differences (*p* < 0.05) among all the pastas for each technological property.

**Table 4:** Pearson’s coefficients for texture parameters (TPA), technological properties, and composition for each pasta studied.

Variables	Hard	Spr	Gum	Frt	M(%)	Octi
**Adh**	−0.965					
**Gum**	0.992					
**Chw**	0.981	0.960	0.992			
**SI(g/g)**				0.992	0.998	
**Prot(%)**						−0.962
**Fdt (%)**						−0.973
**Fsa (%)**						−0.973

Values in bold letters were significant (*p* < 0.05). HAR: Hardness, SPR: Springiness, GUM: Gumminess, FRT: Fracturability, CHW: Chewiness, M: Moisture, OCTI: Instrumental Optimal Cooking Time, SI: Swelling index, PROT: Protein, FDT: Total dietary fiber, FSA: Soluble fiber.

## Data Availability

The datasets generated for this study are available on request to the corresponding author.

## References

[1] Hever J. (2016). Plant-Based Diets: A Physician’s Guide. Perm. J..

[2] EU Commission Novel Food. https://food.ec.europa.eu/safety/novel-food_en.

[3] Fleta Zaragozano J., Fleta Asín J. (2019). Valoración Nutricional y Económica de La Utilización de Algas. Rev. Española Estud. Agrosoc. Pesq..

[4] Cherry P., O’Hara C., Magee P.J., McSorley E.M., Allsopp P.J. (2019). Risks and Benefits of Consuming Edible Seaweeds. Nutr. Rev..

[5] Conchillo A., Valencia I., Puente A., Ansorena D., Astiasarán I. (2006). Componentes Funcionales en Aceites de Pescado y de Alga. Nutr. Hosp..

[6] Roohinejad S., Koubaa M., Barba F.J., Saljoughian S., Amid M., Greiner R. (2017). Application of Seaweeds to Develop New Food Products with Enhanced Shelf-Life, Quality and Health-Related Beneficial Properties. Food Res. Int..

[7] Kadam S.U., Prabhasankar P. (2010). Marine Foods as Functional Ingredients in Bakery and Pasta Products. Food Res. Int..

[8] Veluchamy C., Palaniswamy R. (2020). A Review on Marine Algae and Its Applications. Asian J. Pharm. Clin. Res..

[9] Ródenas P. (2003). Las Algas en la Dieta. Nat. Medicat..

[10] Biernacka B., Dziki D., Miś A., Rudy S., Krzykowski A., Polak R., Różyło R. (2019). Changes in Pasta Properties during Cooking and Short-Time Storage. Int. Agrophys..

[11] Vasiliu M., Navas P.B. (2009). Propiedades de cocción, físicas y sensoriales de una pasta tipo fetuchine elaborada con sémola de trigo durum y harina deshidratada de cebollín (*Allium fistulosum* L.). Saber.

[12] Bruneel C., Pareyt B., Brijs K., Delcour J.A. (2010). The Impact of the Protein Network on the Pasting and Cooking Properties of Dry Pasta Products. Food Chem..

[13] Ficco D.B.M., De Simone V., De Leonardis A.M., Giovanniello V., Del Nobile M.A., Padalino L., Lecce L., Borrelli G.M., De Vita P. (2016). Use of Purple Durum Wheat to Produce Naturally Functional Fresh and Dry Pasta. Food Chem..

[14] Calanche J., Beltrán H., Marquina P., Roncalés P., Beltrán J.A. (2019). Eating Fish in Another Way: Development of Functional Pasta with Added Concentrates of Farmed Sea Bass (*Dicentrarchus labrax*). Cereal Chem..

[15] Ainsa A., Marquina P.L., Roncalés P., Beltrán J.A., Calanche J.B. (2021). Enriched Fresh Pasta with a Sea Bass By-Product, a Novel Food: Fatty Acid Stability and Sensory Properties throughout Shelf Life. Foods.

[16] Lynch J.M., Barbano D.M. (1999). Kjeldahl Nitrogen Analysis as a Reference Method for Protein Determination in Dairy Products. J. AOAC Int..

[17] Mariotti F., Tomé D., Mirand P.P. (2008). Converting Nitrogen into Protein—Beyond 6.25 and Jones’ Factors. Crit. Rev. Food Sci. Nutr..

[18] Asp N.G., Johansson C.G., Hallmer H., Siljeström M. (1983). Rapid Enzymatic Assay of Insoluble and Soluble Dietary Fiber. J. Agric. Food Chem..

[19] Cleary L., Brennan C. (2006). The Influence of a (1→3)(1→4)-*β*-D-Glucan Rich Fraction from Barley on the Physico-Chemical Properties and in Vitro Reducing Sugars Release of Durum Wheat Pasta. Int. J. Food Sci. Technol..

[20] American Association of Cereal Chemists (AACC) (2000). Approved Methods of the American Association of Cereal Chemists.

[21] (2013). de 12 de Septiembre, por el Que se Aprueba la Reglamentación Técnico-Sanitaria para la Elaboración, Circulación y Comercio de Pastas Alimenticias.

[22] Base de Datos BEDCA. http://www.bedca.net/bdpub/.

[23] Gupta S., Abu-Ghannam N. (2011). Recent Developments in the Application of Seaweeds or Seaweed Extracts as a Means for Enhancing the Safety and Quality Attributes of Foods. Innov. Food Sci. Emerg. Technol..

[24] Ainsa A., Roldan S., Marquina P.L., Roncalés P., Beltrán J.A., Calanche Morales J.B. (2022). Quality Parameters and Technological Properties of Pasta Enriched with a Fish by-product: A Healthy Novel Food. Food Process. Preserv..

[25] Rodríguez De Marco E., Steffolani M.E., Martínez C.S., León A.E. (2014). Effects of Spirulina Biomass on the Technological and Nutritional Quality of Bread Wheat Pasta. LWT—Food Sci. Technol..

[26] Vernaza M.G., Biasutti E., Schmiele M., Jaekel L.Z., Bannwart A., Chang Y.K. (2012). Effect of Supplementation of Wheat Flour with Resistant Starch and Monoglycerides in Pasta Dried at High Temperatures: Resistant Starch and Monoglycerides in Pasta. Int. J. Food Sci. Technol..

[27] Ainsa A., Honrado A., Marquina P.L., Roncalés P., Beltrán J.A., Calanche J.B.M. (2021). Innovative Development of Pasta with the Addition of Fish By-Products from Two Species. Foods.

[28] Chang H.C., Wu L.-C. (2008). Texture and Quality Properties of Chinese Fresh Egg Noodles Formulated with Green Seaweed (*Monostroma nitidum*) Powder. J. Food Sci..

[29] Liu T., Hamid N., Kantono K., Pereira L., Farouk M.M., Knowles S.O. (2016). Effects of Meat Addition on Pasta Structure, Nutrition and In Vitro Digestibility. Food Chem..

[30] Aínsa A., Vega A., Honrado A., Marquina P., Roncales P., Gracia J.A.B., Morales J.B.C. (2021). Gluten-Free Pasta Enriched with Fish By-Product for Special Dietary Uses: Technological Quality and Sensory Properties. Foods.

[31] Atwell W.A. (2016). Wheat Flour.

[32] Zen C.K., Tiepo C.B.V., Silva R.V., Reinehr C.O., Gutkoski L.C., Oro T., Colla L.M. (2020). Development of Functional Pasta with Microencapsulated *Spirulina*: Technological and Sensorial Effects. J. Sci. Food Agric..

[33] Monteiro M.L.G., Mársico E.T., Deliza R., Castro V.S., Mutz Y.S., Soares Junior M.S., Caliari M., dos Santos E.A., Conte-Junior C.A. (2019). Physicochemical and Sensory Characteristics of Pasta Enriched with Fish (*Oreochromis niloticus*) Waste Flour. LWT.

[34] Desai A., Brennan M.A., Brennan C.S. (2018). The Effect of Semolina Replacement with Protein Powder from Fish (*Pseudophycis bachus*) on the Physicochemical Characteristics of Pasta. Food Sci. Technol..

[35] Prabhasankar P., Ganesan P., Bhaskar N., Hirose A., Stephen N., Gowda L.R., Hosokawa M., Miyashita K. (2009). Edible Japanese Seaweed, Wakame (*Undaria pinnatifida*) as an Ingredient in Pasta: Chemical, Functional and Structural Evaluation. Food Chem..

[36] Suzuki T., Ohsugi Y., Yoshie Y., Shirai T., Hirano T. (1996). Dietary Fiber Content, Water-Holding Capacity and Binding Capacity of Seaweeds. Fish. Sci..

[37] Cofrades S., López-López I., Solas M.T., Bravo L., Jiménez-Colmenero F. (2008). Influence of Different Types and Proportions of Added Edible Seaweeds on Characteristics of Low-Salt Gel/Emulsion Meat Systems. Meat Sci..

[38] Quitral R.V., Morales G.C., Sepúlveda L.M., Schwartz M.M. (2012). Propiedades nutritivas y saludables de algas marinas y su potencialidad como ingrediente funcional. Rev. Chil. Nutr..

[39] Smatanová N., Lacko-Bartosová M. (2014). Noodle Quality of Winter Wheat Cultivated in Sustainable Farming Systems. J. Cent. Eur. Agric..

[40] Dziki D. (2021). Current Trends in Enrichment of Wheat Pasta: Quality, Nutritional Value and Antioxidant Properties. Processes.

[41] Texture Analysis Properties |Stable Micro Systems. https://www.stablemicrosystems.com/TextureAnalysisProperties.html.

[42] Jyotsna R., Prabhasankar P., Dasappa I., Rao G.V. (2004). Effect of Additives on the Quality and Microstructure of Vermicelli Made from Triticum Aestivum. Eur. Food Res. Technol..

